# Association of a Novel DOCK2 Mutation-Related Gene Signature With Immune in Hepatocellular Carcinoma

**DOI:** 10.3389/fgene.2022.872224

**Published:** 2022-05-10

**Authors:** Yushen Huang, Wen Luo, Siyun Chen, Hongmei Su, Wuchang Zhu, Yuanyuan Wei, Yue Qiu, Yan Long, Yanxia Shi, Jinbin Wei

**Affiliations:** ^1^ Pharmaceutical College, Guangxi Medical University, Nanning, China; ^2^ Department of Gastrointestinal Surgery, The Fourth Affiliated Hospital of Guangxi Medical University, Liuzhou, China

**Keywords:** hepatocellular carcinoma, DOCK2, prognosis, biomarker, immune

## Abstract

Hepatocellular carcinoma (HCC) is a malignant tumor with high morbidity and mortality worldwide. Many studies have shown that dedicator of cytokinesis 2 (DOCK2) has a crucial role as a prognostic factor in various cancers. However, the potentiality of DOCK2 in the diagnosis of HCC has not been fully elucidated. In this work, we aimed to investigate the prognostic role of DOCK2 mutation in HCC. The Cancer Genome Atlas (TCGA) and the International Cancer Genome Consortium (ICGC) cohorts were utilized to identify the mutation frequency of DOCK2. Then, univariate Cox proportional hazard regression analysis, random forest (RF), and multivariate Cox regression analysis were performed to develop the risk score that was significantly related to DOCK2 mutation. Moreover, Gene Set Enrichment Analysis (GSEA), Gene Set Variation Analysis (GSVA), and immune correlation analysis were conducted for an in-depth study of the biological process of DOCK2 mutation involved in HCC. The results revealed that the mutation frequency of DOCK2 was relatively higher than that in non-cancer control subjects, and patients with DOCK2 mutations had a low survival rate and a poor prognosis compared with the DOCK2-wild group. In addition, the secretin receptor (SCTR), tetratricopeptide repeat, ankyrin repeat and coiled-coil domain-containing 1 (TANC1), Alkb homolog 7 (ALKBH7), FRAS1-related extracellular matrix 2 (FREM2), and G protein subunit gamma 4 (GNG4) were found to be the most relevant prognostic genes of DOCK2 mutation, and the risk score based on the five genes played an excellent role in predicting the status of survival, tumor mutation burden (TMB), and microsatellite instability (MSI) in DOCK2 mutant patients. In addition, DOCK2 mutation and the risk score were closely related to immune responses. In conclusion, the present study identifies a novel prognostic signature in light of DOCK2 mutation-related genes that shows great prognostic value in HCC patients; and this gene mutation might promote tumor progression by influencing immune responses. These data may provide valuable insights for future investigations into personalized forecasting methods and also shed light on stratified precision oncology treatment.

## Introduction

Hepatocellular carcinoma (HCC), one of the most common forms of cancer, ranks as the second leading cause of cancer death in the world (Zhao et al., 2018; Roderfeld et al., 2020). At present, the treatments for HCC mainly include liver resection, hepatic transplantation, ablation, and transarterial chemoembolization (TACE) (Chen et al., 2020). Nevertheless, due to the high metastasis and recurrence rate after surgery, the overall prognosis of HCC patients remains poor (Li et al., 2021). Usually, liver cancer is not diagnosed until its advanced stage, which makes the fact that most patients receive either no treatment or only palliative treatment (Mittal et al., 2016), indicating that delayed diagnosis results in low patient survival rates. Although alpha-fetoprotein (AFP) is commonly applied as a tumor indicator for the diagnosis of HCC, its low specificity and accuracy are its shortage, which leads to patients missing the best treatment period (Liang Y. et al., 2021). Thus, there is an urgent need to discover new biomarkers to facilitate early detection and prognostic evaluation of HCC.

Dedicator of cytokinesis 2 (DOCK2), originally known as KIAA0209, encodes CDM protein and has been discovered to be linked with a prognostic factor in various cancers (Chen et al., 2018). Recent research exhibited that a high expression level of DOCK2 conferred a good prognosis of acute myeloid leukemia (Hu et al., 2019). In prostate cancer, many specifically hypermethylated genes were found, including DOCK2, GRASP, HIF3A, and PKFP, among which DOCK2 is the candidate marker with the greatest potentiality (Bjerre et al., 2019). In addition, lower DOCK2 expression was related to a poorer prognosis in colorectal cancer, which was attributed to the regulation of canonical and noncanonical Wnt signaling (Yu et al., 2015). Moreover, the DOCK2 genetic variant caused decreased DOCK2 mRNA transcript levels and might be a prognostic biomarker of non-small-cell lung cancer survival (Du et al., 2021). Notably, the mutation of DOCK2 was discovered to correlate with a high risk of HCC (Huang T. et al., 2021). However, the potentiality of DOCK2 in the diagnosis of HCC has not been fully elucidated.

In this work, we intended to investigate the prognostic role of DOCK2 mutation in HCC. First, The Cancer Genome Atlas (TCGA) and the International Cancer Genome Consortium (ICGC) cohorts were utilized to identify the mutation frequency of DOCK2. After clarifying the characteristic genes that are most related to DOCK2 mutation, the risk score was developed, which played an excellent role in predicting the status of survival, tumor mutation burden (TMB), and microsatellite instability (MSI) in DOCK2 mutant patients. Furthermore, for an in-depth study of the biological processes involved in HCC, Gene Set Enrichment Analysis (GSEA), Gene Set Variation Analysis (GSVA), and immune correlation analysis of DOCK2 were performed. Our findings may identify a novel risk score related to DOCK2 mutation for the prognosis of HCC, contributing to early diagnosis, targeted therapy, and prognostic assessment of HCC.

## Materials and Methods

### Data Processing

In this study, The Cancer Genome Atlas (TCGA, http://cancerge.nome.nih.gov/) (Tomczak et al., 2015) and the International Cancer Genome Consortium (ICGC, www.icgc.org) (Zhang et al., 2019) were used to download somatic mutation data (MAF files) of TCGA-LIHC cohort and the LIRI-JP cohort. The primary objective of the ICGC database was to provide a comprehensive elucidation of genome changes in multiple cancers that result in human disease burden. Among the ICGC database, the tumor data from different cancer types (or subtypes) were collected, including abnormal gene expression, somatic mutation, epigenetic modification, and clinical data. The ICGC database contains 25,000 tumor genomes. Meanwhile, the clinicopathologic characteristics and the prognostic information of the patients in TCGA-LIHC cohort, such as gender, age, and clinical stage, were obtained from the UCSC Xena website (http://xena.ucsc.edu/) (Goldman et al., 2019). Moreover, RNA sequencing data (count value), containing mutation data and survival data of 353 patient samples (TCGA-LIHC), were downloaded from TCGA database for subsequent analysis and were annotated by the annotation file of the GRCh38 version from the Ensembl database (http://ftp.ensembl.org/pub/current_gtf) (Howe et al., 2021). In addition, the copy number variations data were obtained from TCGA database. The clinical characteristics of patients are listed in Table 1.

**TABLE 1:** Summary of patient data sets.

Variable	TCGA set (*n* = 353)	ICGC set (*n* = 258)
Age (years)	—	—
≤55	119	29
>55	234	229
Gender	—	—
Female	116	67
Male	237	191
Histologic grade	—	—
G1	52	39
G2	171	116
G3	113	80
G4	12	23
TNM stage	—	—
I/II	247	N/A
III/IV	85	N/A
Family history	—	—
No	199	164
Yes	106	77

### Mutation Analysis

With the development of tumor genomics, the mutation annotation format (MAF) is being widely accepted and used to store detected somatic mutations. In this study, the maftools package (Mayakonda et al., 2018) and the GenVisR package (Skidmore et al., 2016) were utilized to visualize the somatic mutation data downloaded from TCGA; meanwhile, the GenVisR package was also used to visualize the somatic mutation data obtained from ICGC. Moreover, the mutation of DOCK2 was revealed by the G3viz package (Guo et al., 2019). Additionally, to evaluate whether the genes have copy number variation in liver cancer, GISTIC2.0 in the Genepattern (https://cloud.genepattern.org/) cloud analysis platform was used to analyze the copy number variation data of liver cancer in TCGA database (M. et al., 2006).

### Construction of Dedicator of Cytokinesis 2 Mutation Prediction Model

The liver cancer patients were divided into mutation group (DOCK2-MUT) and wild group (DOCK2-WT) according to the DOCK2 mutation status of the gene expression data downloaded from TCGA. A survival analysis was performed based on the DOCK2 mutation and prognosis information of liver cancer patients, thus investigating the prognostic difference between the DOCK2 mutation group and the wild group. Moreover, the patients’ data obtained from TCGA was randomly divided into a training set (*N* = 264) and a testing set (*N* = 89) at a ratio of 7:3. The DOCK2 mutation prediction model was conducted using the random forest (RF) method (Yperman et al., 2019) in the training set, and the model performance was quantified via the receiver operating characteristic (ROC) curve.

### Construction of the Prognostic Model

The prognostic model was built in light of the gene expression data of 28 DOCK2 mutant liver cancer patients with clinical information. First, univariate Cox proportional hazard regression analysis was performed to initially identify overall survival (OS)-related genes (*p*-value<0.05). Next, RF and multivariate Cox regression analyses were conducted to construct a prognostic model. The formula for calculating the risk score is risk score = exp gene 1 × β gene 1 + exp gene 2 × β gene 2 + exp gene 3 × β gene 3 + … exp gene n × β gene n (exp gene n indicates the expression level of gene n; β gene n indicates the regression coefficient of gene n calculated by multivariate Cox regression). Moreover, correlation analysis was performed between the DOCK2 mRNA expression and the risk score, as well as between the DOCK2 mRNA expression and the characteristic genes mRNA expression in the model.

### Assessment of the Prognostic Model

The liver cancer patients in the DOCK2 mutant group with clinical information were divided into high-risk groups and low-risk groups in light of the median risk score. The OS analysis was performed using the Kaplan–Meier (KM) survival curve and time-dependent ROC, thus evaluating the prediction accuracy of the model. Then, the univariate Cox regression analysis and the multivariate Cox regression analysis were conducted in light of the age, gender, clinical stage, tumor stage, and risk score in DOCK2 mutant liver cancer patients. Meanwhile, the risk score and clinical characteristics were analyzed using correlation analysis.

### Tumor Mutation Burden and Microsatellite Instability Analysis

Given that different DOCK2 mutation types may have different effects on tumorigenesis, the expression data of liver cancer patients were divided into two subgroups: inactivated mutation subgroup and other non-silent mutation subgroups. The two subgroups were assessed *via* the KM survival curve and time-dependent ROC.

Tumor mutation burden (TMB) refers to the total number of somatic mutations in the exon coding region of the genome that have substitutions, insertions, or deletions per Mb base in a tumor sample. The TMB score of each liver cancer sample is the total number of somatic mutations (including non-synonymous point mutations, insertions, and deletions in the coding region of exons)/target region size, and the unit is mutations/Mb (Chan et al., 2019). Microsatellite (MS) is defined as a short tandem repeat (STR) in the human genome including single-nucleotide repeats, dinucleotide repeats, and even more nucleotide repeats; microsatellite instability (MSI) refers to the change of any length of microsatellites due to the insertion or deletion of repeat units in tumor tissues compared to normal tissues (Hile et al., 2013). MSI is calculated as the number of insertions or deletions in gene repeats. In this study, the relationship between the risk score and TMB and the correlation between the risk score and MSI were analyzed, respectively.

### Differential Analysis

In order to assess the impact of gene expression value on the DOCK2 mutant type compared to the DOCK2 wild type, the limma R package (Ritchie et al., 2015) was used to conduct the discrepant analysis between the DOCK2 mutant group and DOCK2 wild-type group. The absolute value of log fold change (logFC) > 0.5 and *p*-value <0.05 were set as the threshold for differentially expressed genes. Among them, the genes with logFC > 0.5 and *p*-value <0.05 were considered upregulated differential genes, while the genes with logFC < −0.5 and *p*-value <0.05 were regarded as downregulated differential genes, and the aforementioned results were displayed with heat maps and volcano maps.

### Gene Functional and Pathway Enrichment Analysis

Gene Ontology (GO) enrichment analysis is a common method for large-scale functional enrichment studies of genes in different dimensions and levels and generally includes three aspects: biological process (BP), molecular function (MF), and cellular component (CC) (Ashburner et al., 2000). The Kyoto Encyclopedia of Genes and Genomes (KEGG) (Ogata et al., 1999) is a widely applied database that stores numerous data about genomes, biological pathways, diseases, and drugs. Additionally, the clusterProfiler R package (Yu et al., 2012) was applied to identify significantly enriched biological processes and pathways by GO functional annotation and KEGG biological pathway enrichment analysis. A *p*-value <0.05 was considered statistically significant.

### Gene Set Enrichment Analysis and Gene Set Variation Analysis

Gene Set Enrichment Analysis (GSEA) is a calculation method to assure whether a set of predefined genes show statistical differences between two biological states, generally applied to estimate changes in the pathway and bioprocess activity in sample expression datasets (Subramanian et al., 2005). Based on the gene expression profile data of DOCK2 mutant group and DOCK2 wild-type group patients in TCGA-LIHC dataset and the reference gene sets “c5.go.v7.4.entrez.gmt” and “c2.cp.kegg.v7.4.entrez.gmt” downloaded from the MSigDB database (Liberzon et al., 2015), the GSEA method included in the clusterProfiler R package was used to conduct enrichment analysis of TCGA-LIHC gene expression profile data, thus studying the differences in the biological processes of genes between the DOCK2 mutant group and DOCK2 wild group. A *p*-value <0.05 was considered statistically significant.

Gene Set Variation Analysis (GSVA) (Hnzelmann et al., 2013; Liberzon et al., 2015), a nonparametric unsupervised analysis method, is widely utilized in the evaluation of metabolic pathways enriched in different samples by converting the expression matrix of genes between different samples into the expression matrix of gene sets between samples. To study the biological process variation of the DOCK2 mutant group compared with the DOCK2 wild group, the “GSVA” R package (Hnzelmann et al., 2013; Liberzon et al., 2015) was used to perform gene set variation analysis, and the enrichment scores of each sample in each pathway in the reference gene set “h.all.v7.4.symbols.gmt” were downloaded from the MSigDB database. Moreover, the GSVA results were also analyzed for correlation with the risk scores.

### Immunoassay

The immune microenvironment is a comprehensive LoAD system, which is mainly composed of immune cells, inflammatory cells, fibroblasts, interstitial tissues, and various cytokines and chemokines. The infiltration analysis of immune cells in tissues has an important guiding role in disease research and treatment prognosis.

ESTIMATE analysis, an algorithm that quantifies the immune activity (immune infiltration level) in tumor samples on the basis of gene expression data, can reflect the richness of the gene characteristics of the matrix and immune cells. The content of stromal cells and immune cells in TCGA-LIHC was calculated by an ESTIMATE R package (Yoshihara et al., 2013). The correlation between the ESTIMATE score and the expression level of characteristic genes and DOCK2 in the prognostic model was also evaluated.

CIBERSORT is an algorithm that deconvolves the expression matrix of immune cell subtypes in light of the principle of linear support vector regression, making use of RNA-Seq data to assess the abundance of immune cells in the tissue. In this study, the proportion of 22 immune cell subtypes in TCGA-LIHC immune microenvironment was calculated by the CIBERSORT algorithm (Newman et al., 2019) in the R package. The number of permutations was 1,000, and a *p*-value <0.05 was considered accurate for calculating the content of immune cells. Based on Pearson correlation analysis, the correlation between the expression levels of characteristic genes and DOCK2 in the prognostic model and 22 types of immune cells in liver cancer was calculated.

To test the biological processes and cell signal transduction pathways that the characteristic genes of the prognostic model may be involved in, an immune gene set was obtained from the ImmPort database (Bhattacharya et al., 2014) (https://www.immport.org), and the relationship between the characteristic genes and DOCK2 in the prognostic model and immune genes was determined. The correlation between the expression of the HLA family and the risk score of the prognostic model was also conducted.

### Statistical Analysis

All data calculations and statistical analysis were performed using R programming (https://www.r-project.org/, version 3.6.3). Multiple testing corrections were determined using the Benjamini–Hochberg (BH) method, and FDR correction was conducted using multiple tests to reduce the false-positive rate. For the comparison of two groups of continuous variables, the statistical significance of normally distributed variables was estimated by independent Student’s t test, and the differences between non-normally distributed variables were analyzed by using the Mann–Whitney U test (the Wilcoxon rank-sum test). The survival R package (Durisová and Dedík, 1993) was utilized in the survival analysis. The Kaplan–Meier survival curve was used to reveal differences in survival, and the significance of the difference in survival time between the two groups of patients was assessed via a log-rank test. Univariate and multivariate Cox analyses were applied to determine independent prognostic factors. As for assessing the accuracy of the risk score to estimate the prognosis, the receiver operator characteristic (ROC) curve was drawn by the pROC package and ROCR package, and the area under the curve (AUC) was calculated (Sing et al., 2005; Robin et al., 2011). All *p*-values reported from statistical tests were two-sided, and a *p*-value <0.05 was considered statistically significant.

## Results

### Genes With High-Frequency Mutations in HCC

**FIGURE 1:**
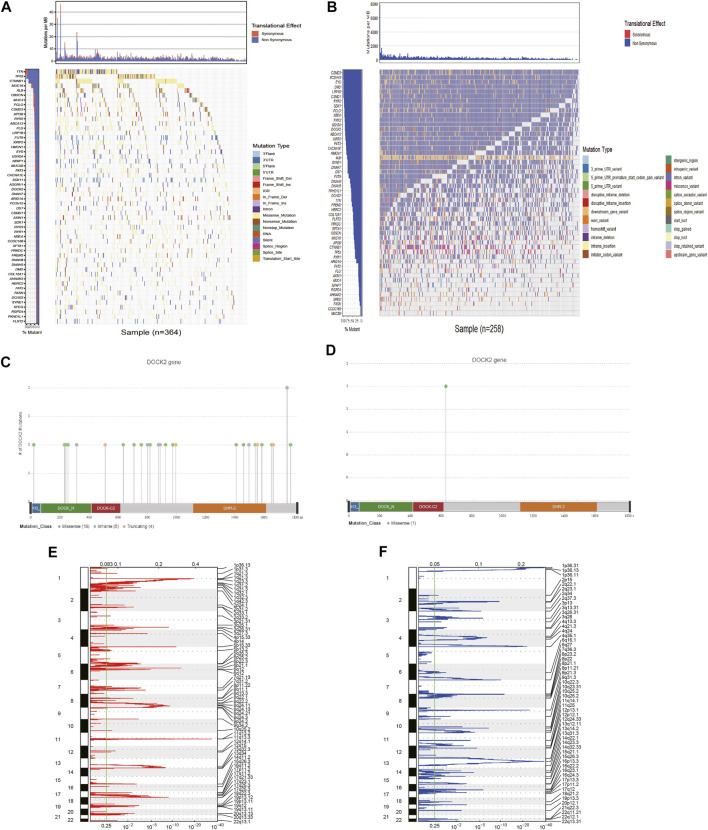
Analysis of somatic mutation and copy number variation in patients with HCC. **(A)** 54 genes with the highest mutation frequency in LIHC patients in TCGA cohort. **(B)** Mutations of 54 genes in ICGC. The panels on the left of the two waterfall charts show genes with high-frequency mutations in different cohorts, and the order was based on their mutation frequency; The panels on the right side of the two waterfall charts reveal different types of mutations represented by various color modules. **(C)** DOCK2 mutation in TCGA cohort. **(D)** DOCK2 mutation in the ICGC cohort. **(E,F)** Identification of significantly amplified and deleted genes. The mRNA located at the focal CNA peak was related to LIHC. The false discovery rate (Q value) and the change score of GISTIC2.0 (x-axis) corresponded to the genome position (y-axis). The dotted line indicates the centromere. The green line represents the significant cutoff (q value of 0.25).

First, 54 genes with mutation frequencies greater than 6% in TCGA-LIHC patients obtained from TCGA were identified (Figure 1A). Moreover, the 54 genes were further confirmed using the data downloaded from the ICGC database (Figure 1B). Among them, the mutation frequency of DOCK2 was relatively high, and the DOCK2 mutation was visualized (Figures 1C, D). GISTIC 2.0 was utilized to analyze copy number variation data in TCGA, identifying obviously amplified or deleted genes, and the results showed that DOCK2 had no significant amplification or deletion (Figures 1E, F).

### Construction of Dedicator of Cytokinesis 2 Mutation Prediction Model

**FIGURE 2:**
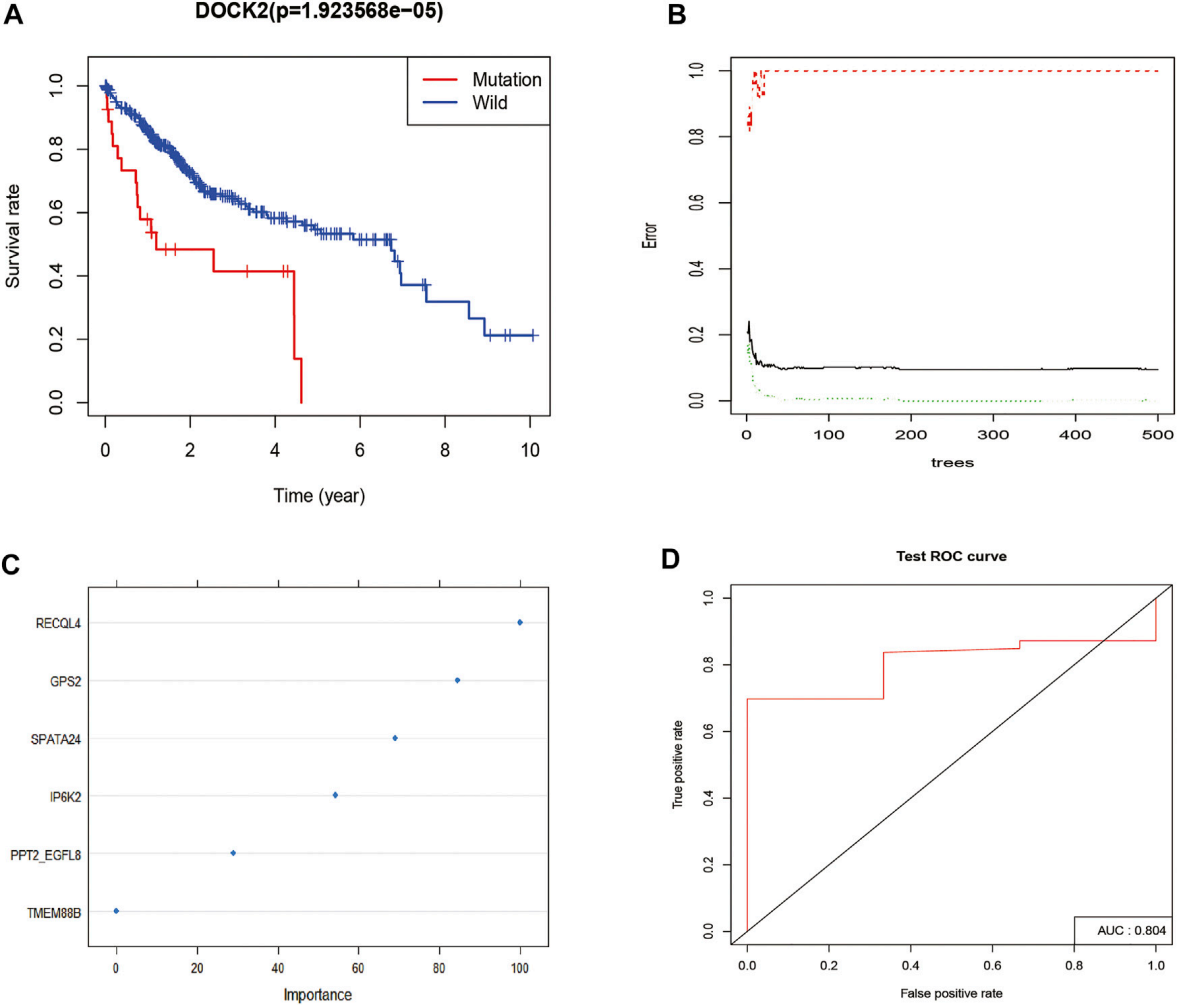
DOCK2 mutation survival analysis and model construction. **(A)** Effect of DOCK2 mutation on OS and its significance. Blue indicates the DOCK2 wild type; red indicates the DOCK2 mutant type. **(B)** Relationship between the model error and the number of decision trees. **(C)** Importance of DOCK2 mutation model variables. **(D)** Performance of the DOCK2 mutation model in the test set.

Survival analysis was conducted based on the DOCK2 mutation data and prognostic information of liver cancer patients, and the results revealed that the mutation of DOCK2 had an essential impact on the prognosis and survival of patients (Figure 2A). In the training set, the RF method was used to construct a DOCK2 mutation prediction model in the mRNA data (Figures 2B, C). The ROC curve was used to evaluate the performance of the model, and AUC scores close to 1 indicated that the model had high sensitivity under a very low false-positive rate. The model AUC value in the training cohort was 1.00 and that in the validation cohort was 80.4% (Figure 2D), which demonstrated that the performance of this model was sufficient to effectively predict DOCK2 mutation in other transcription cohorts.

### Construction of the Prognostic Model

**FIGURE 3:**
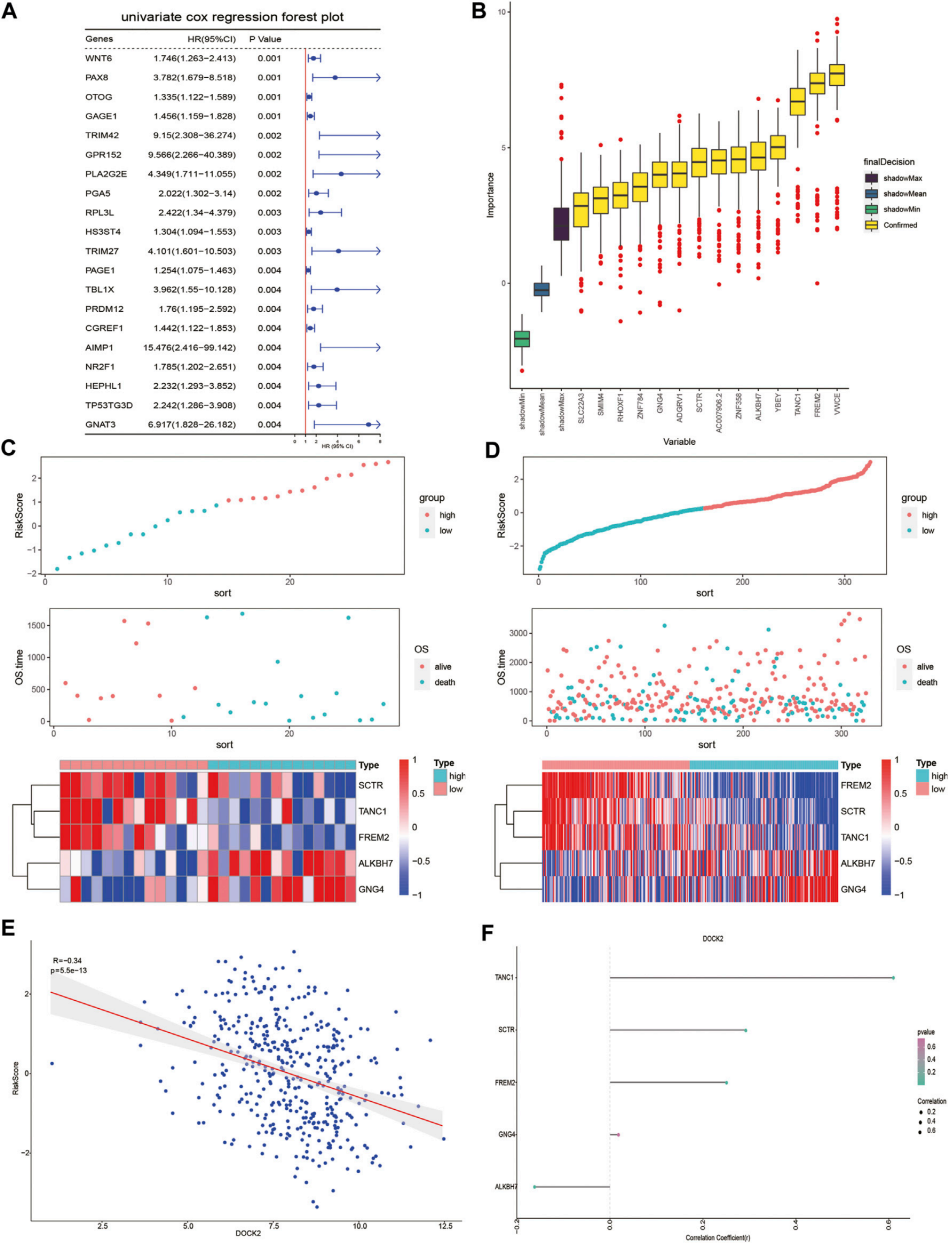
DOCK2 mutation prognostic model. **(A)** Forest plot of the top 20 prognostic-related genes obtained by univariate regression analysis. The left side of the vertical red line is the protective gene, and the right side is the dangerous gene. **(B)** 14 important features selected based on RF. **(C,D)** Risk score, survival status, and characteristic gene expression of DOCK2 mutant and DOCK2 wild type, respectively. **(E)** Scatter plot of the correlation between DOCK2 expression and risk score. **(F)** Correlation between DOCK2 and characteristic genes. The size of the dot represents the strength of the correlation between DOCK2 and the characteristic gene; the size of the point is proportional to the correlation. The color of the dot represents the *p*-value; the greener the color, the smaller the *p*-value, and the pinker the color, the greater the *p*-value. *p*-value ≤ 0.05 was considered statistically significant.

Univariate Cox proportional hazard regression analysis was carried out in the gene expression data of 28 DOCK2 mutant LIHC patients with clinical information and 641 genes related to OS were discovered (*p*-value <0.05) (Figure 3A). Then, we conducted the RF method to find out the most important features connected with prognosis, and 15 genes were screened out (Figure 3B). Finally, a multivariate Cox proportional hazard regression analysis identified the five genes associated with OS, which are secretin receptor (SCTR), tetratricopeptide repeat, ankyrin repeat and coiled-coil domain-containing 1 (TANC1), Alkb homolog 7 (ALKBH7), FRAS1-related extracellular matrix 2 (FREM2), and G protein subunit gamma 4 (GNG4). Cox regression coefficients of the characteristic genes were calculated, and the risk score of each sample was defined as the sum of the expression of each characteristic gene multiplied by its regression coefficient. To assess the predictive power of the prognostic model, the risk scores of DOCK2 mutant and DOCK2 wild-type patients were calculated and ranked, the survival status of each patient was displayed on the dot chart, and the expression of characteristic genes was shown on the heat map (Figures 3C, D). Meanwhile, the correlation between DOCK2 expression and risk score and characteristic gene expression was analyzed, respectively. The expression of DOCK2 was dramatically negatively correlated with the risk score (Figure 3E). DOCK2 expression was significantly positively correlated with SCTR (r = 0.293, *p*-value = 9.6e-10), TANC1 (r = 0.607, *p*-value = 1.8e-43), and FREM2 (r = 0.252, *p*-value = 1.8e-07), whereas the expression of DOCK2 had a significant negative correlation with ALKBH7(r = −0.162, *p*-value = 0.0009) (Figure 3F).

### Assessment of the Prognostic Model

**FIGURE 4:**
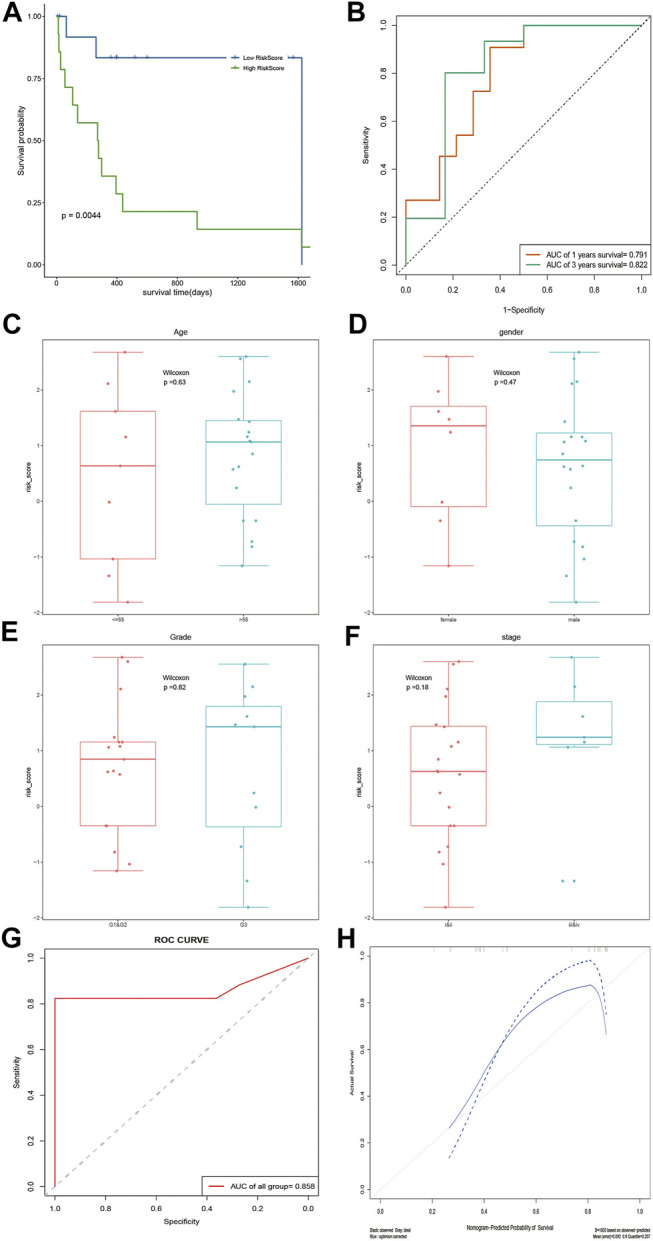
Analysis of the prognostic model and clinical prediction model. **(A)** The impact of risk score on patients’ OS and its significance. Blue meant a low-risk score, and green meant a high-risk score. **(B)** Time-dependent ROC analysis of risk score. **(C–F)** Correlation analysis of risk score with age, gender, tumor stage, and clinical stage. **(G)** ROC curve of a clinical prediction model in 28 DOCK2 mutant samples. **(H)** Calibration curve of the clinical prediction model. The X-axis was the outcome probability predicted by the model. The Y-axis was the value obtained by actual observation, and the calculation was repeated 1,000 times. The blue solid line is the calibration curve, and the diagonal line is the ideal curve. The closer the calibration curve was to the ideal curve, the better the predictive ability of the model.

According to the median risk score, DOCK2 mutant liver cancer patients with clinical information were divided into the high-risk group and low-risk group. The results of survival analysis showed that there was a significant difference in OS between the two risk groups of 28 DOCK2 mutant samples (Figure 4A). The 1- and 3-year AUCs on the basis of the risk score obtained by the prognostic model were 0.791 and 0.822, respectively (Figure 4B). Additionally, the correlation analysis results of the risk score and the clinical characteristics of 28 DOCK2 mutant samples revealed that there were no significant differences in risk scores, different ages, genders, clinical stages, and tumor stages (Figures 4C–F). Then, univariate Cox analysis and multivariate Cox analysis were performed based on the age, gender, clinical stage, tumor stage, and risk score of DOCK2 mutant liver cancer patients, thus building a clinical prediction model, the efficacy of which in 28 DOCK2 mutant samples was 85.8% (Figure 4G). Meanwhile, the calibration curve showed both good discrimination ability and calibration (Figure 4H).

### Tumor Mutation Burden and Microsatellite Instability Analysis

**FIGURE 5:**
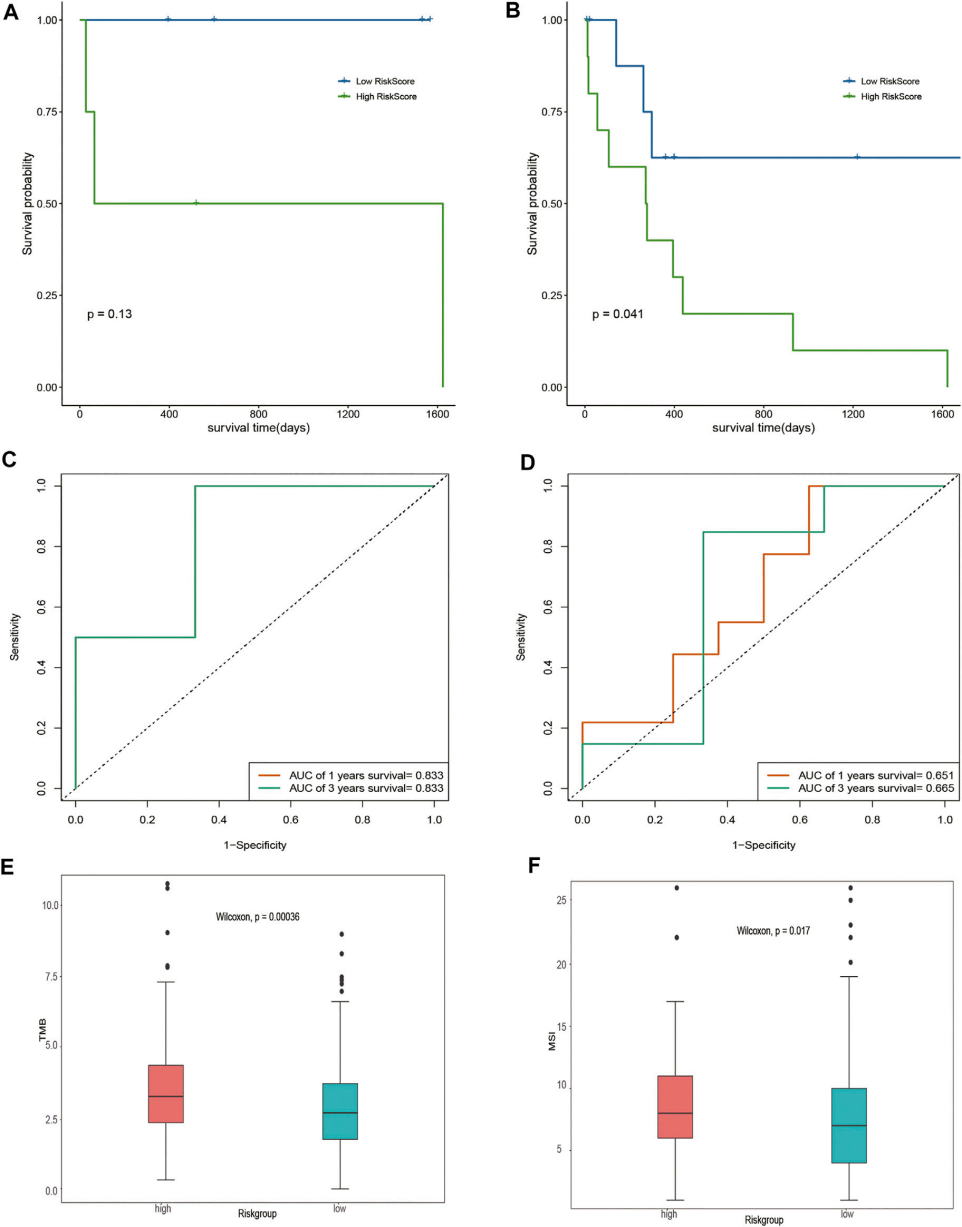
Assessment of risk score. **(A,B)** Impact of risk score on OS in the subgroup of inactivated mutations and other subgroups of non-silent mutations and its significance, respectively. Blue means a low-risk score, and green means a high-risk score. **(C,D)** Time-dependent ROC analysis of the risk score in the subgroup of inactivated mutations and other subgroups of non-silent mutations. **(E)** Analysis of the correlation between TMB and risk score. Pink represents the high-risk group, and green represents the low-risk group. **(F)** Correlation analysis between MSI and risk score. Pink represents the high-risk group, and green represents the low-risk group.

Given that different DOCK2 mutation types may have different effects on the occurrence of liver cancer, this study further divided the gene expression data of 28 DOCK2 mutant LIHC patients into two subgroups: the inactivated mutation subgroup (*n* = 8, containing nonsense mutation and silent mutation) and other non-silent mutation subgroups (*n* = 20). Survival analysis showed that significant differences in OS were observed between the two risk groups of samples in other non-silent mutation subgroups (Figures 5A, B). The time-dependent ROC analysis showed that in the subgroup of inactivated mutations, the 1- and 3-year AUCs of the risk score were both 0.833 (Figure 5C); moreover, in other subgroups of non-silent mutations, the 1- and 3-year AUCs of risk scores were 0.651 and 0.665, respectively (Figure 5D), suggesting that the risk score could still maintain good predictive performance in subgroups with different mutation types. After acquiring the total number of mutations to obtain TMB and assessing the relationship between the risk score and the TMB, we found that there were obvious differences in TMB between samples with different risk scores (*p*-value < 0.05) (Figure 5E). In addition, MSI between samples with different risk scores also had a significant difference (*p*-value < 0.05) (Figure 5F).

### Differential Analysis and Functional Enrichment

**FIGURE 6:**
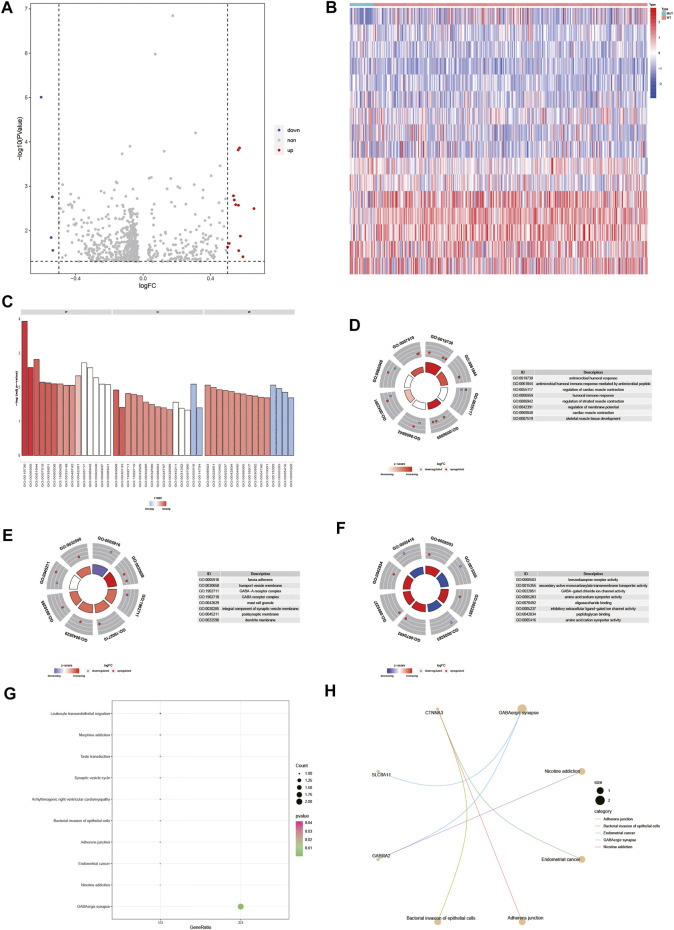
Differential gene and its functional enrichment analysis. **(A)** Abscissa is log2FoldChange, and the ordinate is −log10 (adjust *p*-value). The red nodes indicates upregulation, the blue nodes indicate downregulation, and the gray nodes represent insignificant expression. **(B)** Abscissa is the patient ID, and the ordinate is the differential gene. Red represents high gene expression, and blue represents low gene expression. The green comment bar indicates the DOCK2 mutant sample, while the red comment bar indicates the DOCK2 wild-type sample. **(C–F)** GO function enrichment analysis of differential genes and display of BP, MF, and CC. **(D–F)** Color of the node indicates the level of gene expression value. Blue represents that the expression value was downregulated, and red indicates that the expression value was upregulated. The middle quadrilateral represents the effect of genes on the enriched GO terms. Light color means inhibition; dark color means activation. **(G)** KEGG pathway enrichment analysis. The abscissa is the gene ratio, and the ordinate is the pathway name. The size of the node indicates the number of genes enriched in the pathway, and the color of the node indicates −log10 (*p*-value). **(H)** Display of the first five items in the KEGG enrichment analysis of differential genes.

To analyze the effect of gene expression values on the DOCK2 mutant samples compared with the DOCK2 wild-type samples, we conducted a limma discrepant analysis to obtain differentially expressed genes. The gene expression profile data of 28 DOCK2 mutant samples and 325 DOCK2 wild-type samples were included in TCGA-LIHC, from which 12 upregulated differential genes (*p*-value <0.05, logFC > 0.5) and 4 downregulated differential genes (*p*-value <0.05, logFC < −0.5) were screened out, and the volcanic map and heat map of the differential genes were shown in Figures 6A,B. To determine the value of the differential genes, the biological processes, the cellular components, and the molecular functions were performed. GO functional enrichment analysis was first assessed on the 16 differential genes (Figure 6C and Table 2), and the results showed that these genes were mainly enriched in biological processes such as antimicrobial humoral response, antimicrobial humoral immune response mediated by antimicrobial peptides, regulation of cardiac muscle contraction, humoral immune response, regulation of striated muscle contraction, regulation of membrane potential, cardiac muscle contraction, and skeletal muscle tissue development (Figure 6D); in cellular components including fascia adherens, transport vesicle membrane, GABA-A receptor complex, GABA receptor complex, mast cell granule, integral component of synaptic vesicle membrane, postsynaptic membrane, and dendrite membrane (Figure 6E); and in molecular functions including benzodiazepine receptor activity, secondary active monocarboxylate transmembrane transporter activity, GABA-gated chloride ion channel activity, amino acid:sodium symporter activity, oligosaccharide binding, inhibitory extracellular ligand-gated ion channel activity, peptidoglycan binding, and amino acid:cation symporter activity (Figure 6F). Then, pathways significantly affected by 16 differential genes were also performed (Figure 6G and Table 3), and the data revealed that the 16 differential genes were involved in GABAergic synapse, nicotine addiction, endometrial cancer, adherens junction, and bacterial invasion of epithelial cells (Figure 6H).

**TABLE 2:** GO enrichment analysis.

Ontology	ID	Description	*p*-value
BP	GO:0019730	Antimicrobial humoral response	0.00011696
BP	GO:0061844	Antimicrobial humoral immune response mediated by antimicrobial peptides	0.001532039
BP	GO:0055117	Regulation of cardiac muscle contraction	0.001927973
BP	GO:0006959	Humoral immune response	0.002637965
BP	GO:0006942	Regulation of striated muscle contraction	0.002684261
BP	GO:0042391	Regulation of membrane potential	0.004609488
BP	GO:0060048	Cardiac muscle contraction	0.005272199
BP	GO:0007519	Skeletal muscle tissue development	0.007122358
BP	GO:0035821	Modification of morphology or physiology of other organisms	0.007470229
BP	GO:0060538	Skeletal muscle organ development	0.007915771
BP	GO:1900426	Positive regulation of defense response to bacterium	0.008007218
BP	GO:0006937	Regulation of muscle contraction	0.0080973
BP	GO:0006941	Striated muscle contraction	0.008373123
BP	GO:0033148	Positive regulation of intracellular estrogen receptor signaling pathway	0.008804639
BP	GO:0048742	Regulation of skeletal muscle fiber development	0.008804639
CC	GO:0005916	Fascia adherens	0.008087095
CC	GO:0030658	Transport vesicle membrane	0.012058338
CC	GO:1902711	GABA-A receptor complex	0.015313019
CC	GO:1902710	GABA receptor complex	0.016112846
CC	GO:0042629	Mast cell granule	0.017710673
CC	GO:0030285	Integral component of synaptic vesicle membrane	0.027246665
CC	GO:0045211	Postsynaptic membrane	0.027593411
CC	GO:0032590	Dendrite membrane	0.031982049
CC	GO:0098563	Intrinsic component of synaptic vesicle membrane	0.037479348
CC	GO:0030133	Transport vesicle	0.039367589
CC	GO:0014704	Intercalated disc	0.039826362
CC	GO:0,034,707	Chloride channel complex	0.039826362
CC	GO:0031252	Cell leading edge	0.041396752
CC	GO:0032589	Neuron projection membrane	0.045281879
CC	GO:0097060	Synaptic membrane	0.04693445
MF	GO:0008503	Benzodiazepine receptor activity	0.008670141
MF	GO:0015355	Secondary active monocarboxylate transmembrane transporter activity	0.008670141
MF	GO:0022851	GABA-gated chloride ion channel activity	0.010239011
MF	GO:0005283	Amino acid:sodium symporter activity	0.011022581
MF	GO:0070492	Oligosaccharide binding	0.011805575
MF	GO:0005237	Inhibitory extracellular ligand-gated ion channel activity	0.012587993
MF	GO:0042834	Peptidoglycan binding	0.013369837
MF	GO:0005416	Amino acid:cation symporter activity	0.014151105
MF	GO:0004890	GABA-A receptor activity	0.014931799
MF	GO:0099095	Ligand-gated anion channel activity	0.015711918
MF	GO:0016917	GABA receptor activity	0.017270437
MF	GO:0030552	cAMP binding	0.018048836
MF	GO:0004190	Aspartic-type endopeptidase activity	0.019603918
MF	GO:0005328	Neurotransmitter:sodium symporter activity	0.020380602
MF	GO:0070001	Aspartic-type peptidase activity	0.020380602

**TABLE 3:** KEGG enrichment analysis.

Ontology	ID	Description	*p*-value
KEGG	hsa04727	GABAergic synapse	0.000356208
KEGG	hsa05033	Nicotine addiction	0.014756278
KEGG	hsa05213	Endometrial cancer	0.021348965
KEGG	hsa04520	Adherens junction	0.026092015
KEGG	hsa05100	Bacterial invasion of epithelial cells	0.028275936
KEGG	hsa05412	Arrhythmogenic right ventricular cardiomyopathy	0.028275936
KEGG	hsa04721	Synaptic vesicle cycle	0.028639606
KEGG	hsa04742	Taste transduction	0.031545693
KEGG	hsa05032	Morphine addiction	0.033359051
KEGG	hsa04670	Leukocyte transendothelial migration	0.041671347

### Gene Set Enrichment Analysis and Gene Set Variation Analysis

**FIGURE 7:**
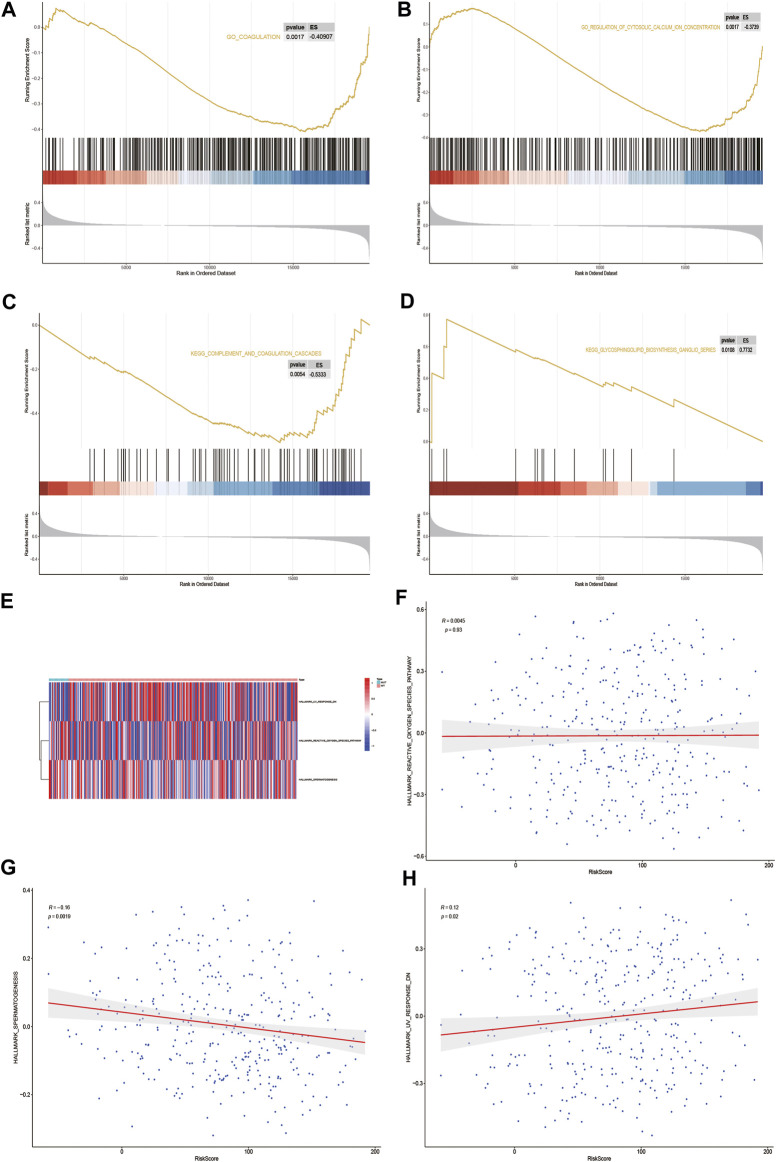
GSEA and GSVA. **(A,B)** Results of GSEA biological function enrichment. **(C,D)** Results of biological pathway enrichment. **(E)** Heat map of the significant hallmark analyzed by GSVA. **(F,H)** Scatter plot of correlation between significant hallmark and risk score.

GSEA biological function enrichment analysis of DOCK2-MUT and DOCK2-WT genes was performed, and the results showed that the genes in DOCK2-MUT and DOCK2-WT were enriched in biological processes including coagulation and regulation of cytosolic calcium ion concentration (Figures 7A, B and Table 4).

**TABLE 4:** GSEA.

ID	Description	ES	*p*-value
go_coagulation	go_coagulation	−0.409069157	0.001672241
go_regulation_of_cytosolic_calcium_ion_concentration	go_regulation_of_cytosolic_calcium_ion_concentration	−0.373853775	0.001692047
go_ventricular_cardiac_muscle_cell_action_potential	go_ventricular_cardiac_muscle_cell_action_potential	−0.635035665	0.001769912
go_cell_cell_adhesion_via_plasma_membrane_adhesion_molecules	go_cell_cell_adhesion_via_plasma_membrane_adhesion_molecules	−0.432720305	0.00177305
go_negative_regulation_of_execution_phase_of_apoptosis	go_negative_regulation_of_execution_phase_of_apoptosis	-0.756145207	0.001785714
go_negative_regulation_of_myoblast_differentiation	go_negative_regulation_of_myoblast_differentiation	−0.729056439	0.001785714
go_regulation_of_execution_phase_of_apoptosis	go_regulation_of_execution_phase_of_apoptosis	−0.642047088	0.001785714
go_regulation_of_inflammatory_response_to_antigenic_stimulus	go_regulation_of_inflammatory_response_to_antigenic_stimulus	-0.726196139	0.001798561
go_negative_regulation_of_synapse_organization	go_negative_regulation_of_synapse_organization	-0.72808206	0.001801802
go_negative_regulation_of_inflammatory_response_to_antigenic_stimulus	go_negative_regulation_of_inflammatory_response_to_antigenic_stimulus	−0.827265127	0.001805054
go_lipid_translocation	go_lipid_translocation	−0.601531891	0.001808318
go_cytokine_receptor_activity	go_cytokine_receptor_activity	−0.540903843	0.001828154
go_blood_microparticle	go_blood_microparticle	-0.531068688	0.001838235
go_homophilic_cell_adhesion_via_plasma_membrane_adhesion_molecules	go_homophilic_cell_adhesion_via_plasma_membrane_adhesion_molecules	−0.531976492	0.001848429
go_complement_activation	go_complement_activation	−0.566062924	0.001865672
go_immune_receptor_activity	go_immune_receptor_activity	−0.526876463	0.001872659
go_organophosphate_ester_transport	go_organophosphate_ester_transport	−0.455964352	0.001879699
go_dna_replication_independent_nucleosome_organization	go_dna_replication_independent_nucleosome_organization	0.584583627	0.002118644
go_odorant_binding	go_odorant_binding	0.524374367	0.002132196
go_digestion	go_digestion	0.456798178	0.002145923
go_diencephalon_development	go_diencephalon_development	0.542992829	0.002159827
go_ear_morphogenesis	go_ear_morphogenesis	0.482732218	0.002164502
go_neuron_fate_commitment	go_neuron_fate_commitment	0.623987975	0.002164502
go_endocrine_system_development	go_endocrine_system_development	0.486005649	0.002169197
go_appendage_morphogenesis	go_appendage_morphogenesis	0.489850317	0.002178649
go_keratinization	go_keratinization	0.454250476	0.002178649
go_cornification	go_cornification	0.531790615	0.002188184
go_embryonic_skeletal_system_development	go_embryonic_skeletal_system_development	0.503417996	0.002188184
go_skeletal_system_morphogenesis	go_skeletal_system_morphogenesis	0.440722934	0.002188184
go_embryonic_appendage_morphogenesis	go_embryonic_appendage_morphogenesis	0.475526703	0.002192982
go_cell_fate_specification	go_cell_fate_specification	0.504467657	0.002197802
go_divalent_inorganic_anion_homeostasis	go_divalent_inorganic_anion_homeostasis	0.825664161	0.002202643
go_embryonic_skeletal_system_morphogenesis	go_embryonic_skeletal_system_morphogenesis	0.578909723	0.002207506
go_anterior_posterior_pattern_specification	go_anterior_posterior_pattern_specification	0.475479352	0.002222222
go_proximal_distal_pattern_formation	go_proximal_distal_pattern_formation	0.65212998	0.002227171
go_appendage_development	go_appendage_development	0.473257146	0.002247191
go_cornified_envelope	go_cornified_envelope	0.629843229	0.002252252
go_hindlimb_morphogenesis	go_hindlimb_morphogenesis	0.642853948	0.002252252
go_negative_regulation_of_response_to_extracellular_stimulus	go_negative_regulation_of_response_to_extracellular_stimulus	0.795788231	0.002252252
go_muscle_cell_fate_commitment	go_muscle_cell_fate_commitment	0.824992566	0.002277904
go_neuron_fate_specification	go_neuron_fate_specification	0.68805324	0.00228833
go_cell_fate_commitment	go_cell_fate_commitment	0.400964549	0.002309469
go_monovalent_inorganic_anion_homeostasis	go_monovalent_inorganic_anion_homeostasis	0.667695897	0.002309469
go_sensory_organ_morphogenesis	go_sensory_organ_morphogenesis	0.430365367	0.002309469
go_embryonic_organ_morphogenesis	go_embryonic_organ_morphogenesis	0.424037318	0.002364066
kegg_complement_and_coagulation_cascades	kegg_complement_and_coagulation_cascades	−0.533293095	0.001824818
kegg_glycosphingolipid_biosynthesis_ganglio_series	kegg_glycosphingolipid_biosynthesis_ganglio_series	0.773222168	0.004329004
kegg_propanoate_metabolism	kegg_propanoate_metabolism	−0.574271735	0.018248175
kegg_renin_angiotensin_system	kegg_renin_angiotensin_system	−0.660325041	0.023897059
kegg_natural_killer_cell_mediated_cytotoxicity	kegg_natural_killer_cell_mediated_cytotoxicity	−0.4049327	0.024822695
kegg_ribosome	kegg_ribosome	0.412873966	0.041942605
kegg_peroxisome	kegg_peroxisome	−0.435572819	0.042628774
kegg_primary_bile_acid_biosynthesis	kegg_primary_bile_acid_biosynthesis	−0.639717566	0.043478261
kegg_hematopoietic_cell_lineage	kegg_hematopoietic_cell_lineage	−0.426295539	0.045207957

Next, the results of GSEA biological pathway enrichment analysis suggested that biological pathways such as complement and coagulation cascades, glycosphingolipid biosynthesis, and ganglio series were identified among the targets in DOCK2-MUT and DOCK2-WT (Figures 7C, D and Table 4).

Furthermore, in order to comprehensively evaluate the roles of the targets in DOCK2-MUT and DOCK2-WT in liver cancer, the GSVA was conducted. The data showed three hallmarks: reactive_oxygen_species_pathway, spermatogenesis, and uv_response_dn (Figure 7E). Among them, spermatogenesis was significantly negatively correlated with risk score (*p*-value <0.05); uv_response_dn was obviously positively related with risk score (*p*-value <0.05); however, reactive_oxygen_species_pathway had no significant correlation with risk score (Figures 7F–H).

### Immunoassay

**FIGURE 8:**
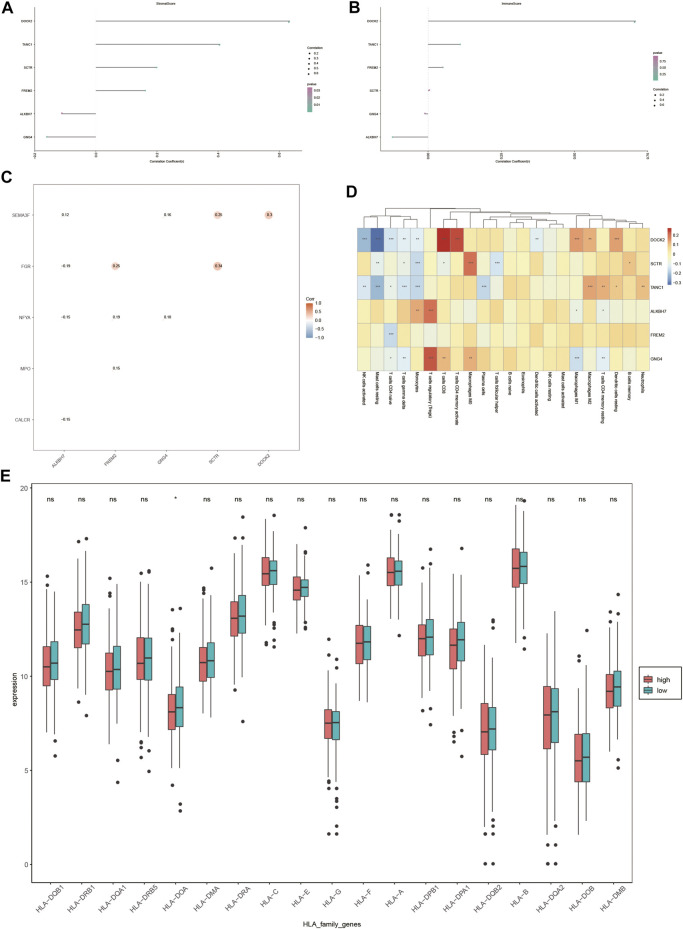
Immune correlation analysis. **(A,B)** Correlation of DOCK2 and characteristic genes with the content of immune cells and stromal cells. **(C)** Correlation between DOCK2 and characteristic genes and immune genes. **(D)** Correlation of DOCK2 and characteristic gene expression with immune cell infiltration. **(E)** Correlation between HLA family expression and the risk score.

As liver cancer is considered an immunogenic tumor, the relationship between the expression of DOCK2, SCTR, TANC1, ALKBH7, FREM2, and GNG4 and the levels of immune cells and stromal cells was assessed (Figures 8A, B). The data showed a positive correlation between stromal cells and DOCK2, SCTR, TANC1, and FREM2, and a negative correlation between stromal cells and ALKBH7 and GNG4. Moreover, immune cells had a positive correlation with DOCK2 and TANC1 and a negative correlation with ALKBH7 (*p*-value <0.05).

In addition, the six target genes, DOCK2, SCTR, TANC1, ALKBH7, FREM2, and GNG4, were significantly correlated with specific immune-related genes. For example, DOCK2 was significantly related to the immune gene of SEMA3F; SCTR was correlated with SEMA3F and FGR; and the same situation occurred between GNG4 and SEMA3F and NFYA, FREM2 and FGR, NFYA and MPO, ALKBH7 and SEMA3F, FGR, NFYA and CALCR (*p*-value <0.05) (Figure 8C).

More importantly, the six target genes were obviously interrelated with the infiltration of numerous immune cells. DOCK2 gene expression was distinctly related to the infiltration of 11 immune cells; SCTR gene expression was dramatically correlated with the infiltration of 7 immune cells; TANC1 gene expression was obviously interrelated with the infiltration of 10 immune cells; FREM2 gene expression was distinctly related to the infiltration of one immune cell; ALKBH7 gene expression was dramatically correlated with the infiltration of 4 immune cells; GNG4 gene expression was markedly interrelated with the infiltration of 7 immune cells (*p*-value <0.05) (Figure 8D). Furthermore, the expression value of HLA-DOA was statistically significant in different risk groups (Figure 8E).

## Discussion

It is worth noting that genetic mutation plays an essential role in HCC. Some reports showed that the genetic mutation of some important genes, including TP53, CTNNB1, and AXIN1, was relevant to poor outcomes for patients with HCC (Zhan et al., 2013; Schulze et al., 2015). It is suggested that exploring genomic instability is a great way to discover promising prognostic biomarkers for the treatment of HCC. DOCK2 has been discovered to be linked with a prognostic factor in various cancers such as acute myeloid leukemia, prostate cancer, colorectal cancer, and non-small-cell lung cancer (Du et al.; Yu et al., 2015; Bjerre et al., 2019; Hu et al., 2019). Nevertheless, research on the diagnosis ability of DOCK2 in HCC remains insufficient. In the present study, a high mutation of DOCK2 was found in TCGA-LIHC cohort, which was further verified in the LIRI-JP cohort, indicating that DOCK2 mutation was significantly frequent in HCC. Moreover, survival analysis showed that patients with DOCK2 mutation had a low survival rate and a poor prognosis compared with the DOCK2 wild-type group, suggesting that DOCK2 might exhibit a great value in the prognosis of HCC.

Given the frequency of DOCK2 mutation in HCC, it is essential to conduct an in-depth study of an effective method for predicting the prognosis of DOCK2 mutant HCC patients. Thus, we calculated the risk score on the basis of the five most relevant prognostic genes including SCTR, TANC1, ALKBH7, FREM2, and GNG4. The risk score exhibited great predictive ability in different DOCK2 mutation statuses, risks, and types. Moreover, the risk score showed an excellent correlation with TMB and MSI. Moreover, the clinical prediction model based on age, gender, clinical stage, tumor stage, and risk score revealed both good discrimination ability and calibration, suggesting that these clinical features could independently predict the prognosis of patients with HCC. In addition to providing prognostic information, these five genes can also be used in precise oncology as biomarkers to guide targeted therapy.

SCTR, encoding the protein named G protein-coupled receptor, belongs to the glucagon–VIP–secretin receptor family (Bayliss and Starling, 1902). It has been reported that in colorectal cancer, hypermethylation of SCTR had a diagnostic value (Li et al., 2020). Moreover, SCTR was also found to be a predictor of the risk for breast cancer and pancreatic ductal adenocarcinoma (Zheng et al., 2018; Park et al., 2020). TANC1 has an ankyrin repeat (AR) domain that participates in many cell functions, especially tumorigenesis (Yang et al., 2019). Through Ingenuity Pathway Analysis (IPA), genes regulated by TANC1 were enriched in hepatic inflammation and HCC (Wu et al., 2021). ALKBH7, a mitochondrial ketoglutarate dioxygenase, decreases ROS formation to regulate programmed necrosis (Meng et al., 2019; Kulkarni et al., 2020). A single-nucleotide polymorphism (SNP) of ALKBH7 was clarified as a new prostate cancer biomarker in 2017 (Walker et al., 2017). FREM2 belongs to an extracellular matrix protein located in the dense layer of the epithelial basement membrane (Wang et al., 2021). In prostate adenocarcinoma, FREM2 was found to be one of the most recurrently mutated genes (Zhao et al., 2019). Upregulated FREM2 protein expression was demonstrated in glioblastomas compared to normal samples (Jovcevska et al., 2019). GNG4 is one of the fourteen γ-subunit proteins of the G protein-coupled receptor (Kishibuchi et al., 2020). As a tumor suppressor gene, abnormal expression of GNG4 was reported in multiple cancers containing colorectal cancer, bladder cancer, and glioblastoma (Pal et al., 2016; Zhang et al., 2018; Liang L. et al., 2021). To sum up, evidence has shown that the five genes clarified in this work all have essential roles in malignant development, indicating that developing corresponding targeted therapies for high-risk DOCK2-mutant HCC was feasible.

To understand the role of DOCK2 mutation in HCC from multiple angles, its potential mechanism in this disease should be focused on. Through the analysis of GO, KEGG, GSEA, and GSVA, we found that DOCK2 mutation could influence humoral immune response, transport vesicle membrane, mast cell granule, adherens junction, complement and coagulation cascades, and reactive oxygen species pathway. More importantly, these biological processes and pathways are closely correlated with immune function. Immunity plays an essential role in tumor development including tumor proliferation, invasion, and metastasis. A significantly important reason for tumor initiation and progression is that the tumor microenvironment (TME) changes from immune activation to immune suppression, thereby avoiding immune surveillance (Han et al., 2019). In addition, increasing evidence showed that genetic mutation was not adequate to start tumors, and TME acted as the second hit that might be needed to drive tumor development (Sahoo et al., 2018). The TME consists of the stromal and immune cells (Huang H. et al., 2021). Both stromal cells and immune cells were found to be significantly correlated with DOCK2 and the characteristic genes of the prognostic model, indicating that DOCK2 might regulate the immune process to promote the development of HCC. There are many immune cells involved in tumorigenesis and progression. For the in-depth investigation, SEMA3F, FGR, NFYA, MPO, and CALCR showed a high correlation with DOCK2 and its characteristic genes. In addition, the expression level of HLA-DOA revealed a significant difference in different DOCK2 risk groups. Thus, the six immune genes, namely, SEMA3F, FGR, NFYA, MPO, CALCR, and HLA-DOA, might be the targets of DOCK2 immune-related treatments in the future.

Although the current work sheds new light on the relationship between DOCK2 and HCC, there were still some limitations. First of all, the number of cohorts with both TCGA-LIHC and LIRI-JP was restricted, and multi-center large sample research is needed. Second, given that the data were obtained from public resources, the bias of the analyzed profile could not be ignored. Finally, all the results in this work came from *in silico* analyses, and further clinical validations and experiments are required to promote the clinical application of our findings, which will be our next research content in the near future.

In conclusion, the present study identifies a novel prognostic signature based on DOCK2 mutation-related genes that shows great prognostic value in HCC patients, and this gene mutation might promote tumor progression by influencing immune responses. These data provide valuable insights for future investigations into personalized forecasting methods and also shed light on stratified precision oncology treatment.

## Data Availability

The datasets presented in this study can be found in online repositories. The names of the repository/repositories and accession number(s) can be found in the article/Supplementary Material.
